# Intestinal cholesterol embolism resulting from intra-aortic balloon pumping: a case report

**DOI:** 10.1186/1752-1947-8-213

**Published:** 2014-06-20

**Authors:** Satoshi Yamaguchi, Masanori Kakazu, Arasaki Osamu

**Affiliations:** 1 Department of Cardiology, Tomishiro Central Hospital, 25 Ueta, Tomishiro-shi, Okinawa, Japan

**Keywords:** Intra-aortic balloon pumping, Cholesterol embolism, Intestinal ischemia, Intestinal necrosis

## Abstract

**Introduction:**

Intra-aortic balloon pumping is used in elective percutaneous coronary intervention for increasing coronary blood flow. However, intra-aortic balloon pumping may decrease visceral blood flow and cause mesenteric ischemia by visceral artery obstruction.

**Case presentation:**

We report the case of a 79-year-old Asian man in whom elective percutaneous coronary intervention was performed with intra-aortic balloon pumping. He died from mesenteric ischemia 25 hours after the procedure. Microscopic findings showed that intra-aortic balloon pumping had detached the aortic plaque, breaking it into systemic emboli, leading to subsequent intestinal ischemia and necrosis.

**Conclusions:**

We conclude that intra-aortic balloon pumping can cause an intestinal cholesterol embolism.

## Introduction

In intra-aortic balloon pumping (IABP), an intra-aortic balloon is inserted percutaneously and placed in the descending aorta. Its counterpulsation contributes to systolic unloading and diastolic augmentation. However, IABP can cause a systemic cholesterol embolism. Therefore, there is a debate as to whether the elective use of IABP during a high-risk percutaneous coronary intervention (PCI) has a beneficial or detrimental effect on mortality and morbidity [1]. We describe a case of mesenteric cholesterol embolism during the use of IABP.

## Case presentation

A 79-year-old Asian man presented with a transient right hemiplegia and recovered within 24 hours, after which he experienced sudden dyspnea and desaturation. An echocardiography revealed left ventricular anterior wall hypokinesis. There was no ST-T change on the electrocardiogram. A coronary angiography revealed three-vessel disease and left main coronary artery disease. He did have a history of congestive heart failure, which was treated with the administration of a heparin infusion and nitrate isosorbide.

During rehabilitation, he experienced severe chest pain, and ST-T elevation was detected in leads V2-V5. We planned PCI with IABP support for the treatment of his left main coronary artery lesion.

**Figure 1:**
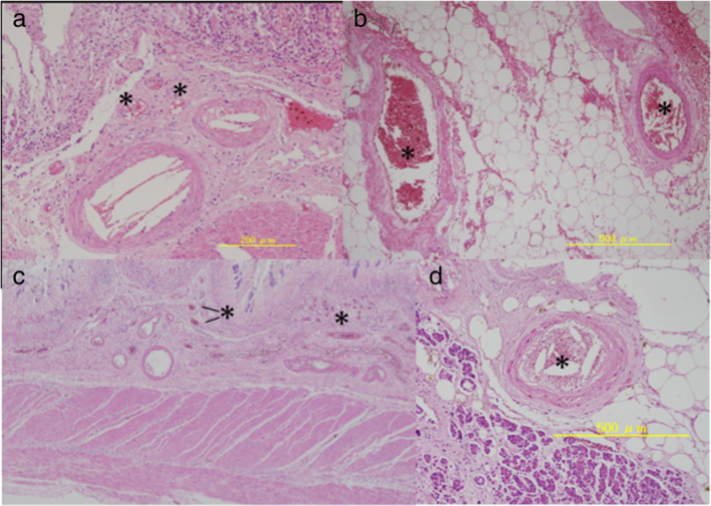
**Cholesterol emboli in (a) the intestinal muscularis propria, (b) the intestinal tela subserosa, (c) the duodenum, and (d) the spleen.** Asterisks (*) show the cholesterol embolisms.

ST-T depression in leads V2-V5 and severe hypokinesis in left ventricular anterior wall suggested myocardial ischemia, thus, the intra-aortic balloon was placed appropriately before PCI. PCI was performed with IABP without any complication. IABP was continued after PCI for cardiac support. However, after the procedure, he experienced uncontrollable metabolic acidosis and anemia with a distended abdomen. He died 25 hours after PCI.His autopsy revealed a massive ischemia in the small intestine, with necrosis in some parts. Microscopic findings revealed cholesterol emboli in the necrotic parts of the small intestine (Figure 1).

## Discussion

**Figure 2:**
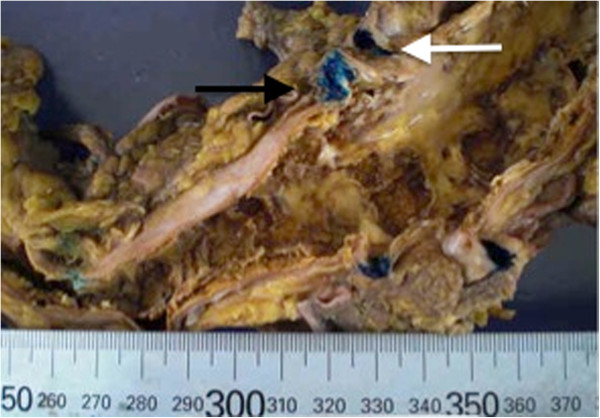
**Abdominal aortic plaque.** Black arrow indicates the supra mesenteric artery. White arrow indicates the celiac artery.

The aortic plaque in our patient was massive and fragile (Figure 2). Microscopic findings indicated that IABP detached the aortic plaque and broke it into small particles, which spread as cholesterol emboli within 24 hours. To the best of our knowledge this time course was faster than any previously reported [2,3]. The emboli spread to the systemic arterioles (Figure 1), including those supplying the small intestine, leading to intestinal ischemia and necrosis.

An abdominal ultrasonography scan may be useful for the detection of intestinal ischemia. As intestinal ischemia may be associated with IABP it is preferable to switch to another form of cardiac support, such as catecholamines or percutaneous cardiopulmonary support, rather than continue IABP, although it is difficult to discontinue IABP when the patient is in a state of cardiogenic shock similar to that experienced in our patient.

A previous study reported that selective IABP for elective PCI has no effect on the mortality and morbidity [1]. Using routine IABP with severe coronary stenosis is not recommended.

## Conclusions

Cholesterol emboli, which were caused by the use of IABP and resulted in mesenteric ischemia, were observed in the microscopic findings upon our patient’s autopsy.

## Consent

Written informed consent was obtained from the patient's next of kin for publication of this case report and accompanying images. A copy of the written consent is available for review by the Editor-in-Chief of this journal.

## Abbreviations

IABP: Intra-aortic-balloon pumping; PCI: Percutaneous coronary intervention.

## Competing interests

The authors declare that they have no competing interests.

## Authors’ contributions

SY wrote this manuscript. MK gave clinical advice as an attending staff member. AO allowed this case to be published. All authors read and approved the final manuscript.

## References

[B1] Perera D Stables R Thomas M Booth J Pitt M Blackman D de Belder A Redwood S BCIS-1 Investigators Elective intra-aortic balloon counterpulsation during high-risk percutaneous coronary intervention JAMA 2010 304 867 874 10.1001/jama.2010.1190 20736470

[B2] Tierney G Parissis H Baker M Austin D Clelland C Richens D An experimental study of intra aortic balloon pumping within the intact human aorta Eur J Cardiothorac Surg 1997 12 486 493 10.1016/S1010-7940(97)01205-0 9332931

[B3] Perler BA Vascular complications of intra-aortic balloon counterpulsation Arch Surg 1983 118 957 10.1001/archsurg.1983.01390080059015 6870525

